# Neuroprotective Activity of Methanolic Extract of *Lysimachia christinae* against Glutamate Toxicity in HT22 Cell and Its Protective Mechanisms

**DOI:** 10.1155/2020/5352034

**Published:** 2020-01-13

**Authors:** Gahee Ryu, Choong Je Ma

**Affiliations:** ^1^Department of Medical Biomaterials Engineering, College of Biomedical Science, Kangwon National University, Chuncheon 24341, Republic of Korea; ^2^Institute of Bioscience and Biotechnology, Kangwon National University, Chuncheon 24341, Republic of Korea

## Abstract

**Purpose:**

Excessive glutamate amount can give oxidative stress to neuronal cells, and the accumulation of cell death can trigger the neurodegenerative disorders. In this study, we discovered the neuroprotective effect of *Lysimachia christinae* Hance in the mouse hippocampal HT22 cell line.

**Method:**

Overnight incubated HT22 cells were pretreated with *L. christinae* extract dose dependently (1, 10, and 100 *μ*g/ml). Followed by then, glutamate was treated. These treated cells were incubated several times again, and cell viability, accumulation of reactive oxygen species (ROS) and Ca^2+^, mitochondrial membrane potential (MMP), and glutathione-related enzyme amount were measured.

**Results:**

As a result, *L. christinae* increases the cell viability by inhibiting the ROS and Ca^2+^ formation, recovering the level of MMP and enhancing the activity of glutathione production compared with only vehicle-treated groups.

**Conclusion:**

These draw that *L. christinae* may remarkably decelerate the neurodegeneration by minimizing neuronal cell damage via oxidative stress.

## 1. Introduction

Year after year, the aging population goes on increasing rapidly, and due to the extended average life expectancy, neurodegenerative disorders become the serious conversation topic. Neurodegeneration arouses the continuous and irrevocable death of neuronal cells, and this causes the defect of cognitive and motor ability [1]. Alzheimer's disease, Parkinson's disease, Huntington disease, and dementia are the typical cases that provoked by neurodegenerative disorder [2]. Numerous medications for neuronal diseases that constituted by chemical compounds exist, but their remedial effect is not sufficient and their side effects become the serious flaws. Therefore, many research studies for screening the undiscovered natural products that have excellent neuroprotective effect are undertaking [3–5].

The pathogenesis that leads to neurodegeneration is not fully recovered, but generally, excitotoxicity, oxidative stress, and mitochondrial dysfunction become one of the reasons for this symptom [6, 7]. Glutamate triggers the pathological neuronal cell death by excitotoxicity, and this presumed to be mediated by reactive oxygen species (ROS) [8–11]. The glutamate toxicity can be classified by two types: receptor-mediated toxicity [12] and nonreceptor-mediated toxicity [13]. Oxidative glutamate toxicity is firstly occurred by high contents of extracellular glutamate which blocks the cystine inflow to the neuronal cells via the cystine/glutamate antiporter system, and this is followed by the lack of intracellular cysteine and glutathione. The depletion of the intracellular glutathione leads to accumulation of ROS resulting in cellular injury. Accumulation of excess ROS brings about receptor-mediated cellular toxicity. It evokes the overexpression of the ionotropic Ca^2+^ receptor [14]. The collapse of Ca^2+^ homeostasis brings about the decline of mitochondrial membrane potential that finally gives rise to mitochondrial malfunction [15].

*Lysimachia christinae* Hance grows naturally in temperate climates and can be easily found in various regions of China [16, 17]. It contains many chemical compounds like flavonoids, triterpenoids, and glucopyranosides [18]. *L. christinae* was diversely used as Chinese traditional medicines as a remedy for cholecystitis and cholagogic effects [19]. Followed by recent studies, dieresis and hepatoprotective and antihyperlipidemic activities of *L. christinae* are additionally proved by the scientific method [20–22]. It also turned up the antioxidant and anti-inflammatory activity [23–25].

HT22 cell has been generally used for the model of *in vitro* experiment to recover the glutamate-triggered oxidative toxicity in the neuronal cell [26, 27]. This cell line is defective in the ionotropic glutamate receptor. Therefore, many research studies that aim at the glutamate-induced cell death can be performed through stimulation of oxidative stress [28]. According to these backgrounds, the neuroprotective activity of *L. christinae* against glutamate toxicity was disclosed for the first time, and its protective mechanisms were revealed by receptor-mediated and nonreceptor-mediated methods.

## 2. Methods and Materials

### 2.1. Plant Material

The dried whole plants of *L. christinae* were bought from the health functional food market named Chunjigayakcho (Seoul, Korea). 6.0 kg of *L. christinae* were extracted by the ultrasonication method three times in 80% methanol for 90 min. The leftover solvent from extracted solution was evaporated by the rotary decompressed evaporator. This voucher specimen named CJ156 M has been stored in the natural product laboratory in Kangwon National University (Chuncheon, Korea).

### 2.2. Cell Culture

Mouse hippocampal-derived HT22 cells were granted by Seoul National University (Seoul, Korea). Dulbecco's modified Eagle's medium (DMEM) was bought from Sigma Aldrich, and fetal bovine serum (FBS) was bought from Gibco BRL Co. (U.S.A). HT22 cells were cultivated in DMEM retaining 2 mg/ml of NaHCO_3_, 15 mM of HEPES, and 1% penicillin/streptomycin with 10% (v/v) FBS under the condition of 37°C humidified atmosphere containing 5% CO_2_. After overnight incubation, these cultured cells were seeded in two different methods—at a density of 2.0 × 10^4^ cells/well in 48 well plates for cell viability, ROS, and Ca^2+^ and MMP measurements and at a density of 3.4 × 10^4^ cells/well in 6 well plates for glutathione-related test.

### 2.3. Cell Viability Test

HT22 cells were incubated one day before the experiment in a 48-well culture plate followed by the above method. Cell viability test was aimed at two different considerations. One is to evaluate the own toxicity of *L. christinae* in the cell, and the other is to evaluate the protective effect against glutamate toxicity. In the self-toxicity test, *L. christinae* was treated in the concentration of 1, 5, 10, 20, 50, and 100 *μ*g/ml. To assess the neuroprotective effect of *L. christinae*, the pretreatment was performed with or without *L. christinae* an hour before glutamate treatment. 6-Hydroxy-2,5,7,8-tetramethylchroman-2-carboxylic acid (Trolox) was used as a positive control material. Whole wells were treated with 2.5 mM glutamate after pretreatment, except for the control group. After one more overnight incubation, the survival rate of cells in both experiments was measured by the 3-(4,5-dimethylthiazol-2-yl)-2,5-diphenyltetrazoliumbromide (MTT) assay. The absorbance was read by a microplate reader at 540 nm. The percentage of surviving cells was expressed relative to the control values.

### 2.4. Measurement of Reactive Oxygen Species (ROS) in the Cell

Intracellular ROS amount was evaluated by using 2,7-dichlorofluorescein diacetate (H_2_DCF-DA) (Invitrogen, U.S.A), which was melted in Hanks' balanced salt solution (HBSS). The 48-well plates passing through the same pretreatment process were used. After 8 h incubation with 2.5 mM glutamate, the cells were added with 10 *μ*M H_2_DCF-DA for 30 min. The stained cells were washed with PBS and suspended in 1% Triton X-100. The result was analyzed for fluorescence intensity excited at 485 nm and emitted at 528 nm using the fluorometer.

### 2.5. Measurement of Intracellular Ca^2+^ Influx

Intracellular Ca^2+^ influx was evaluated by using Fura-2AM. Cells were seeded and pretreated same as ROS measuring assay, but 20 *μ*M of Fura-2 AM was additionally applied. An hour after pretreatment, cells were added with 2.5 mM glutamate and incubated for 2 hours. Subsequently, cells were washed with PBS and suspended in 1% Triton X-100. Ca^2+^-dependent fluorescence intensity was analyzed excited at 340 nm and emitted at 380 nm using the fluorometer.

### 2.6. Measurement of Mitochondrial Membrane Potential (Δ*ψ*_m_)

Mitochondrial membrane potential (MMP) was evaluated by using rhodamine 123 (Rho-123). Cells that were seeded and pretreated identically with the earlier stage were used. After 24 h incubation with 2.5 mM glutamate, the cells were stained by10 *μ*M Rho 123 for 30 min. Cells were washed through PBS and melted with 1% Triton X-100. The result revealed as fluorescence intensity was analyzed under 485 nm excitation wavelength and 528 nm emission wavelength using the fluorometer.

### 2.7. Preparation before Glutathione-Related Test

6-well plate cultured cells were used in the estimation of total glutathione amount. After 24 h incubation, cells were passed through the same pretreatment and treatment process as cell viability test. After 24 h, incubated cells were centrifuged at 3000 g for 30 min at 4°C. The supernatant was collected and put into 96 well plates for per 300 *μ*l. These plates were used in the glutathione-related test—measurement of total glutathione (GSH + GSSG) amount, glutathione peroxidase (GPx) activity, and glutathione reductase (GR) activity.

### 2.8. Estimation of Total Intracellular Glutathione

Total glutathione (GSH + GSSG) amount in the supernatants of cells was estimated according to the enzymatic cycling method by Kim [29]. The 96-well plates prepared at the previous process were used in the following three different experiments. 0.3 mM NADPH, 0.6 mM DTNB (5,5′-dithiobis-2-nitrobenzoic acid, Ellman's reagent), and glutathione disulfide reductase (GSSG-R) 5 unit/ml were put into the whole wells. After 30 seconds incubation for reaction, the result was evaluated by absorbance read at 312 nm using the microplate reader.

### 2.9. Estimation of Glutathione Peroxidase (GPx) and Glutathione Reductase (GR) Activity

GPx activity was evaluated by the following the method of [30]. 0.4 mM NADPH, 0.2 mM H_2_O_2_, 1 mM of L-glutathione reduced (GSH), and 1 unit/ml of glutathione disulfide reductase (GSSG-R) were added to the supernatants laid at 96-well plates. Following then, the changed concentration of NADPH was read by the microplate reader at 340 nm. GR activity was evaluated followed by the method of [31]. 0.1 mM NADPH and 1 mM oxidized glutathione (GSSG) were added to all the reactants. After 2 minutes incubation for reaction, the absorbance was immediately read at 340 nm using the microplate reader.

### 2.10. Chemical Profile of *L. christinae* Extract

*L. christinae* extract was analyzed by HPLC-DAD. HPLC (Dionex) was composed of an LPG 3X00 pump, an ACC-3000 autosampler, a DAD-3000 (RS) diode array UV/VIS detector, and a column oven. Each sample was injected and isolated through a Dionex C18 column (5 *μ*m, 120 Å, 4.6 mm × 150 mm) at 25°C. The mobile phase consisted of 0.1% TFA water and acetonitrile. The injection volume of samples was 30 *μ*l. The UV wavelength was 205, 254, 280, and 330 nm, respectively, and the chromatograms were acquired at 205 nm.

### 2.11. Statistical Analysis

The whole of the experiments was replicated at least three times. Values were expressed as mean ± standard deviation (S.D), and statistical significances were decided by one-way analysis of variance (ANOVA) along with Tukey's test. Values of ^*∗*^*p* < 0.05, ^*∗∗*^*p* < 0.01, and ^*∗∗∗*^*p* < 0.001 were accepted to be statistically significant. Cell experiment data were expressed as relative % setting control group on 100%.

## 3. Results

### 3.1. Protective Effect of *L. christinae* in HT22 Cells against Glutamate-Induced Toxicity

**Figure 1:**
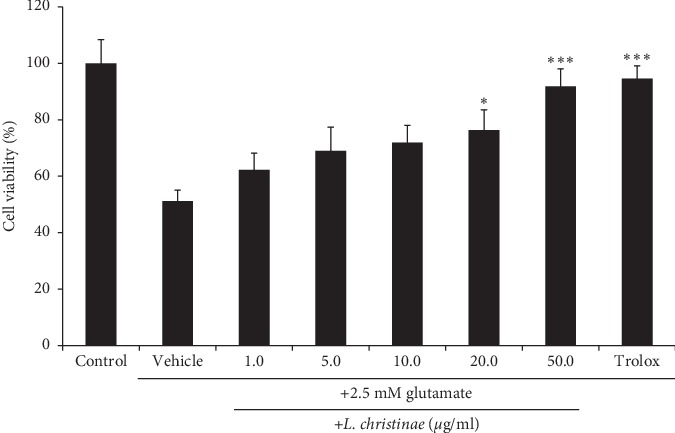
Cell viability after treatment of *L. christinae* extract and glutamate in HT22 cells. Cells were pretreated with 1.0, 5.0, 10.0, 20.0, and 50.0 *μ*g/ml of *L. christinae* and trolox (30 *μ*M), respectively. Then, 2.5 mM glutamate was treated for 30 *μ*l after 1 h. Each bar expresses the mean ± S.D of three replicated processes. ^*∗*^*p* < 0.05, ^*∗∗*^*p* < 0.01, and ^*∗∗∗*^*p* < 0.001 compared to glutamate-injured cells (ANOVA).

To measure the cytotoxicity of the *L. christinae* in HT22 cells, pretreatment was firstly performed at the concentration of 1, 5, 10, 20, and 50 *μ*g/mL. The results showed that *L. christinae* up to 50 *μ*g/mL for 24 h did not express the significant cytotoxic effects (data not shown). To investigate whether *L. christinae* protects the HT22 cells from glutamate-mediated neuronal cell death, the cells were added with or without 2.5 mM glutamate and with or without *L. christinae* at concentrations under 50 *μ*g/mL. As a result, *L. christinae* dose dependently attenuated the cell death (Table 1 and Figure 1). Especially in the highest concentration, the cell survived about 85.69%, close to 100%. Based on these data, succeeding experiments used 10, 20, and 50 *μ*g/mL of *L. christinae*.

### 3.2. Inhibitory Effect of *L. christinae* on Intracellular ROS Production

**Figure 2:**
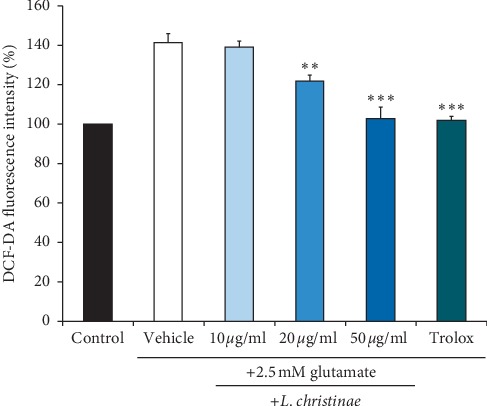
The ROS level of *L. christinae* against glutamate toxicity in HT22 cells. Cell was pretreated with 10, 20, and 50 *μ*g/ml of *L. christinae* and trolox (30 *μ*M). Then, 2.5 mM glutamate was treated for 30 *μ*l after 1 h. Each bar expresses the mean ± S.D of three replicated processes. ^*∗*^*p* < 0.05, ^*∗∗*^*p* < 0.01, and ^*∗∗∗*^*p* < 0.001 compared to glutamate-injured cells (ANOVA).

The activity of enough antioxidant may supplement the oxidative stress caused by ROS. Therefore, we examined whether *L. christinae* can work as an antioxidant inhibiting the glutamate-induced ROS production. Intracellular ROS levels were increased by treatment with 2.5 mM glutamate compared with untreated control cells. In cells treated with 50 *μ*g/ml *L. christinae*, glutamate treatment slightly increased intracellular ROS levels compared with untreated control cells (Figure 2). These results suggested that *L. christinae* may play a role as an effective antioxidant in the neuronal cell.

### 3.3. Inhibitory Effect of *L. christinae* on Intracellular Ca^2+^ Influx

**Figure 3:**
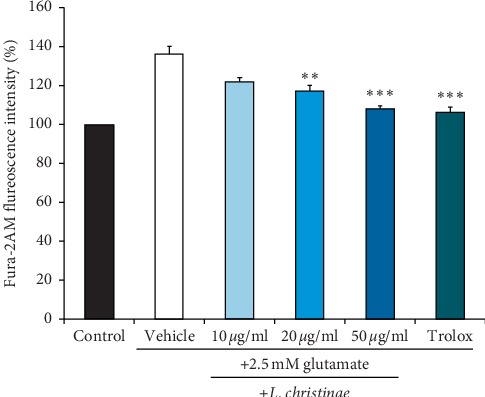
Ca^2+^ influx of *L. christinae* against glutamate toxicity in HT22 cells. Cell was pretreated with 10, 20, and 50 *μ*g/ml of *L. christinae* and trolox (30 *μ*M). Then, 2.5 mM glutamate was treated for 30 *μ*l after 1 h. Each bar expresses the mean ± S.D of three replicated processes. ^*∗*^*p* < 0.05, ^*∗∗*^*p* < 0.01, and ^*∗∗∗*^*p* < 0.001 compared to glutamate-injured cells (ANOVA).

Although the exact mechanisms were not clearly revealed, it is known that oxidative stress induced by glutamate destroys Ca^2+^ homeostasis, and this depolarizes the membrane of mitochondria. Therefore, we investigate the intracellular Ca^2+^ level in HT22 cells after treatment of *L. christinae*. The level of Ca^2+^ influx which is presented by Fura-2 AM dramatically decreased at 20 and 50 *μ*g/mL (Figure 3). The fluorescence intensity of Ca^2+^ level decreased from 127.30% to 112.77% and 108.92% at 10 *μ*g/mL, 20 *μ*g/mL, and 50 *μ*g/mL perspective. All of the results have high significance toward the negative control.

### 3.4. Protective Effect of *L. christinae* against Glutamate-Induced Mitochondrial Depolarization

**Figure 4:**
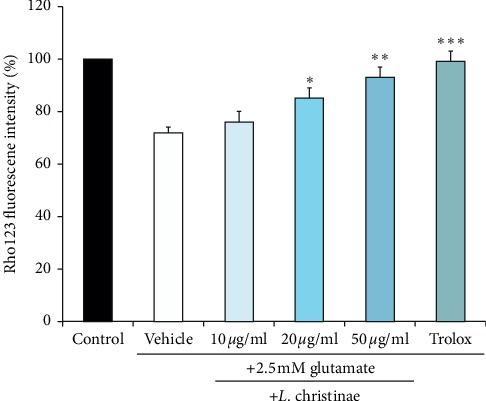
The mitochondrial membrane potential level of *L. christinae* against glutamate toxicity in HT22 cells. Cell was pretreated with 10, 20, and 50 *μ*g/ml of *L. christinae* and trolox (30 *μ*M). Then, 2.5 mM glutamate was treated for 30 *μ*l after 1 h. Each bar expresses the mean ± S.D of three replicated processes. ^*∗*^*p* < 0.05, ^*∗∗*^*p* < 0.01, and ^*∗∗∗*^*p* < 0.001 compared to glutamate-injured cells (ANOVA).

As mentioned above, glutamate toxicity can finally arouse to mitochondrial membrane destruction. To evaluate whether *L. christinae* protects the mitochondrial depolarization, the level of mitochondrial membrane potential was measured. The collapsed mitochondrial membrane potential presents the mitochondrial damage. Rho123 aggregates in the normal state of mitochondria, but in the apoptotic depolarized cell, it is diffused from the cell and releases a green fluorescence. Therefore, if the mitochondrial membrane of the cell has been protected, the fluorescence intensity increases. As shown in Figure 4, *L. christinae* treatment fairly augmented the fluorescence intensity. It showed strongest fluorescence at the highest concentration with the value of 91.85% while 74.97% at the glutamate group.

### 3.5. Effect of *L. christinae* on Total Amount of Intracellular Glutathione

**Figure 5:**
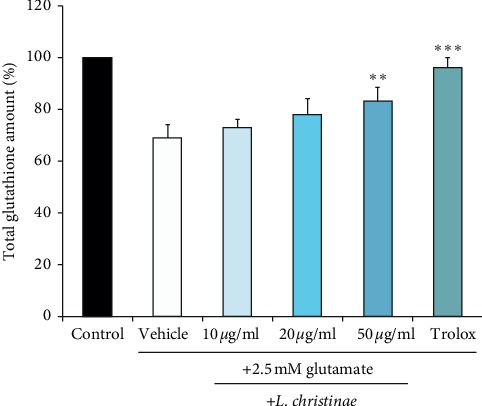
The total glutathione amount of *L. christinae* against glutamate toxicity in HT22 cells. Cell was pretreated with 10, 20, and 50 *μ*g/ml of *L. christinae* and trolox (30 *μ*M). Then, 2.5 mM glutamate was treated for 30 *μ*l after 1 h. Each bar expresses the mean ± S.D of three replicated processes. ^*∗*^*p* < 0.05, ^*∗∗*^*p* < 0.01, and ^*∗∗∗*^*p* < 0.001 compared to glutamate-injured cells (ANOVA).

The sulfide group of reduced glutathione (GSH) reacts with DTNB and altered to a yellow-colored TNB. GSTNB, the mixed disulfide form, recycles glutathione and makes more TNB. In other words, the total amount of the TNB product presents the proportion of total contents of glutathione (GSH + GSSG). By using this principle, total amount of intracellular glutathione was measured. Glutathione was plenty enough at the concentration of 50 *μ*g/ml with the value of 83.59% compared to those treated with only glutamate, 69.92% (Figure 5).

### 3.6. Enhancing Effect of *L. christinae* on Glutathione-Related Enzyme Activities

**Figure 6:**
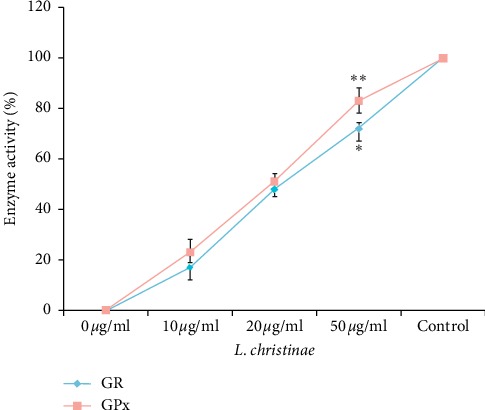
The enzyme activity of GPx and GR when treated with *L. christinae* against glutamate toxicity in HT22 cells. Cell was pretreated with 10, 20, and 50 *μ*g/ml of *L. christinae* and trolox (30 *μ*M). Then, 2.5 mM glutamate was treated for 30 *μ*l after 1 h. Each spot expresses the mean ± S.D of three replicated processes. ^*∗*^*p* < 0.05, ^*∗∗*^*p* < 0.01, and ^*∗∗∗*^*p* < 0.001 compared to glutamate-injured cells (ANOVA).

Glutathione peroxidase (GPx) oxidizes GSH to GSSG and glutathione reductase (GR) reduces GSSG to GSH. The activity of GPx was evaluated by detecting the content of oxidized GSSG, and the activity of GR was evaluated by reduced rate of GSSG in the presence of NADPH. GPx significantly showed activity after 50 *μ*g/mL of 73.27% when compared with the glutamate group. The activity of GR increased about 81.22% at 50 *μ*g/mL statistically *p* < 0.01 significance (Figure 6).

### 3.7. Chemical Profile of the *L. christinae* Extract

**Figure 7:**
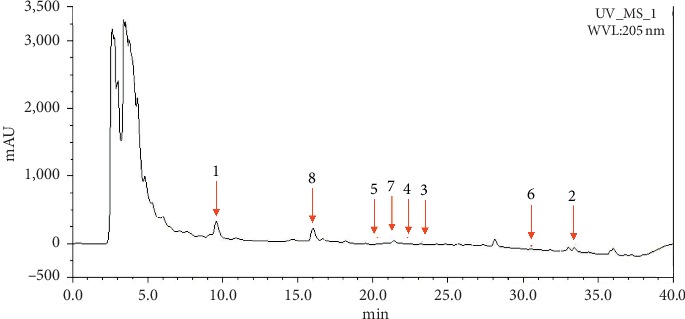
Chemical profile of the *L. christinae* extract.

Eight compounds, cynaroside (1), androst-16-ene-3,6-diol (2), 2-hydroxy-24-propoxy-4-tetracosenoic acid (3), 2-hydroxy-24-methoxy-4-tetracosenoic acid (4), and stearylester ricinoleic acid (5) were isolated from butanol fraction, and *β*-sitosterol (6), (E)-4-(3,4-dimethoxyphenyl)but-3-en-1-yl palmitate (7), and 2-(3,4-dimethoxyphenyl)ethylO-*α*-L-arabinopyranosyl-(1 ⟶ 2)-O-[6-deoxy-*α*-L-mannopyranosyl-(1 ⟶ 3)]-*β*-D-glucopyranoside (8) were identified in the *L. christinae* extract by HPLC-DAD analysis (Figure 7). HPLC chromatogram of the *L. christinae* extract is shown Figure 7. Among them, cynaroside and androst-16-ene-3,6-diol were showed potent neuroprotective activity in a dose-dependent manner. We plan to elucidate which component has an important role to exert neuroprotective activity of the *L. christinae* extract by more research.

## 4. Discussion

To this day, although many researchers struggle to investigate the exact mechanism of neurodegeneration, it has not been unearthed. Though there are some potential reasons for accounting the onset and progression of neurological diseases, one of the reasons is connected to oxidative stress induced by excitotoxicity [32]. Glutamate, the major excitatory neurotransmitter, reduces the uptake of cystine via the glutamate/cystine antiporter, and low level of cystine contributes to decrease in the synthesis of glutathione [33]. Low level of glutathione cannot activate the glutathione redox cycle which is engaged with glutathione peroxidase (GPx) and glutathione reductase (GR). This mechanism is classified into nonreceptor-mediated radical stress. Due to the deficient glutathione, intracellular ROS cannot be effectively eliminated, and this causes the receptor-mediated radical stress [34]. The oxidative stress goes along to downstream phases including the Ca^2+^ concentration increment through the receptor, reduction of mitochondrial membrane potential, malfunction of mitochondria, and finally to cell apoptosis [35].

The above mechanisms propose that targeting the intracellular ROS elimination is a key strategy for developing the effective neuroprotective agent. We firstly found the *L. christinae* extract remarkably enhanced the cell viability against glutamate toxification in HT22 cells according to the concentration. To elucidate how *L. christinae* protects the neuronal cell line, several additional experiments were performed. For identifying the receptor-mediated pathway, ROS and Ca^2+^ production and MMP level were measured. *L. christinae* extract significantly decreased the ROS and Ca^2+^ amount in neuronal HT22 cells. By this result, we could draw the conclusion that *L. christinae* blocked the ROS influx through the cellular receptor, and this continued to block the Ca^2+^ channel. Moderate concentration of Ca^2+^ could not collapse the mitochondrial membrane, so MMP level was increased in the *L. christinae*-treated group. For identifying the nonreceptor-mediated pathway, intracellular glutathione amount and the activities of glutathione redox cycle enzymes were investigated. *L. christinae* addition increased the total GSH/GSSG amount in cell and significantly activated the function of GPx and GR. *L. christinae* may compensate for the loss of antioxidant enzymes such as glutathione, and the elevated level of GSH recovered the glutathione metabolism pathway. The newly regenerated glutathione via the redox cycle directly detoxified the produced ROS.

By this way, *L. christinae* 80% methanol extract conspicuously protected the neuronal HT22 cell against glutamate toxicity. According to these results, *L. christinae* has possibility to be a novel neuroprotective agent toward radical-mediated neuronal cell death. Because *L. christinae* is easily consumed as health functional herbal tea and widely distributed in the moderate climate area, it can be the inexpensive resources with less side effects. Further *in vivo* experiments and structural identification should be conducted afterward. In this study, protective effect of *L. christinae* in neuronal HT22 cells against glutamate excitotoxicity and its protection mechanisms were elucidated for the first time. *L. christinae* exerted remarkable neuroprotective effect in the HT22 cell. It enhanced the activity of antioxidant enzymes such as GPx and GR, so intracellular glutathione amount was increased. Also, *L. christinae* itself works as an antioxidant, so scavenging the ROS activity creates the synergy effect. Reduced ROS maintained Ca^2+^ homeostasis and prevented the collapse of MMP. By these mechanisms, *L. christinae* noticeably attenuated the glutamate-induced oxidative stress.

## 5. Conclusions

From these data, we suggested that the *Lysimachia christinae* extract showed neuroprotective activity against glutamate-injured HT22 cells. Also, this activity was associated with antioxidative activity of the *L. christinae* extract.

## Figures and Tables

**Table 1:** Protective ability of *L. christinae* against glutamate toxicity in HT22 cells.

Groups	Relative protection (%)
Control	100
Glutamate	0
Trolox	93.03 ± 1.91^*∗∗∗*^
*L. christinae*	
1 *μ*g/ml	1.71 ± 3.27
5 *μ*g/ml	3.54 ± 2.18
10 *μ*g/ml	10.98 ± 2.30
20 *μ*g/ml	42.94 ± 4.12^*∗*^
50 *μ*g/ml	85.69 ± 3.60^*∗∗∗*^

## Data Availability

The data used to support the findings of this study are available from the corresponding author upon request.
